# Hypoglycaemia in severe malaria, clinical associations and relationship to quinine dosage

**DOI:** 10.1186/1471-2334-10-334

**Published:** 2010-11-22

**Authors:** Gilbert N Ogetii, Samuel Akech, Julie Jemutai, Mwanamvua Boga, Esther Kivaya, Greg Fegan, Kathryn Maitland

**Affiliations:** 1 Centre for Geographic Medicine Research, Kenya Medical Research Institute- Wellcome Trust Programme, PO Box 230, Kilifi, Kenya; 2 Centre for Clinical Vaccinology and Tropical Medicine, Nuffield Department of Clinical Medicine, University of Oxford, Oxford, UK; 3 Department of Paediatrics and Wellcome Trust Centre for Clinical Tropical Medicine, Faculty of Medicine, Imperial College, Norfolk Place, London, W2 1PG, UK

## Abstract

**Background:**

Hypoglycaemia is an independent risk factor for death in severe malaria and a recognized adverse treatment effect of parenteral quinine. In 2006 our hospital changed quinine treatment policy from 15 mg/kg loading (plus 10 mg/kg 12-hourly) to 20 mg/kg loading (plus 10 mg/kg 8-hourly) to comply with new WHO guidelines. This presented us with the opportunity to examine whether there was any dose relationship of quinine and hypoglycaemia occurrence.

**Methods:**

Retrospective case notes review of all children admitted to hospital with severe falciparum malaria between April 2002 - July 2009, before and after the introduction of the new WHO quinine regimen. Four-hourly bedside glucose levels were measured until intravenous quinine was discontinued. Clinical events immediately preceding or concurrent with each episode of hypoglycaemia (glucose < = 3.0 mmol/l) were recorded.

**Results:**

954 children received the old quinine regime and 283 received the new regime. We found no evidence of an increased prevalence of hypoglycaemia (< = 3.0 mmol/L) on the new regime compared to former (15% vs. 15%); similar findings were noted for profound hypoglycaemia (< 2.2 mmols/L) 8% v 5%, P = 0.07. Episodes were co-incident with disease severity markers: coma (57%), circulatory failure (38%) and respiratory distress (21%) but less commonly with seizures (10%). Disruption of maintenance fluids and/or blood transfusion concurred with 42% of the hypoglycaemia episodes. Post admission hypoglycaemia increased odds of fatal outcome (24%) compared to euglycaemic counterparts (8%), odds ratio = 3.45 (95% confidence interval = 2.30-5.16) P < 0.01.

**Conclusion:**

There was no evidence to indicate a dose relationship between quinine and occurrence of hypoglycaemia. Hypoglycaemia concurred with severity features, disruption of glucose infusion and transfusion. Careful glucose monitoring should be targeted to these complications where resources are limited.

## Background

Hypoglycaemia is an important complication of severe *Plasmodium falciparum *malaria, particularly in children and pregnant women. The prevalence of hypoglycaemia appears to vary in different parts of the world and in different age groups ranging from 8% in SE Asian adults [1] to 10-20% in African children [2-5]. Hypoglycaemia in children is independently associated with poor outcome [6-8] and an increased mortality [2,9-11] predominantly when accompanied by acidaemia (pH < 7.3) or hyperlactataemia (lactate > 5 mmol/l) [12,13].

Hypoglycaemia occurs both on presentation to hospital and during the course of admission in children with severe malaria. The aetiology is incompletely understood and is likely to be multifactorial. Depletion of glucose stores due to starvation, parasite utilisation of glucose, cytokine-induced impairment of gluconeogenesis have been implicated [14]. Hyperinsulinaemia, secondary to quinine therapy, has been advanced as an iatrogenic cause and is well established in adults [15,16]. Data on its relationship in African children with severe malaria are relatively few, but what does exist indicates that hyperinsulinaemia rarely accompanies hypoglycaemia either at admission or during quinine treatment [3,17]. However, considerable concern still exists and intravenous dosing is strongly recommended in solutions containing dextrose to avert the risk of hypoglycaemia[18].

There have been no studies that have explored dose dependency of quinine and the occurrence of hypoglycaemia. In 2006, Kilifi District Hospital (KDH) adopted the new World Health Organization (WHO) guidelines for parenteral quinine treatment of severe falciparum malaria, which recommended 20 mg/kg loading dose of quinine followed by 10 mg/kg 8-hourly [18]. Our previous regime, based on pharmacokinetic studies by Winstanley et al [19,20], initiated treatment with a 15 mg/kg loading dose followed by 10 mg/kg twice daily had been in used since 1998. The objective of this study was to examine the relationship of quinine dosage and occurrence of hypoglycaemia by examining the frequency of hypoglycaemia in children treated with either of the two regimes. We investigated clinical events and factors temporally associated with the development of hypoglycaemia during the course of admission.

## Methods

The study entailed a retrospective review of case notes for two groups of children (0-12 years) with severe malaria admitted to the paediatric high dependency unit (HDU) of Kilifi District Hospital that is run by Kenya Medical Research Institute/Wellcome Trust Programme, Kenya. All paediatric admissions have a standard admission proforma completed and baseline laboratory investigations. All children presenting for admission to hospital, are triaged, to identify those with life-threatening complications and those who comply with the definition of severe malaria are promptly transferred following stabilisation to our high dependency unit. Parental consent for clinical descriptive surveillance or audit is obtained for all infant paediatric admissions. Ethical approval for ongoing surveillance has been granted by the Kenya Medical Research Institute (KEMRI) ethics committee. The case notes of unselected children fulfilling strictly-defined criteria for severe malaria (*P. falciparum *parasitaemia plus impaired consciousness and/or respiratory distress)[6] were reviewed for this study. Impaired consciousness was defined as Blantyre coma score (BCS) < = 2, or prostration (inability to breast feed if < 9 months, or inability to sit unsupported if > = 9 months). Respiratory distress identified children with deep 'Kussmaul' breathing, a clinical feature of metabolic acidosis and an independent risk factor for fatal outcome [6]. Mid Upper Arm circumference (MUAC) < 12 cm, visible severe wasting and/or the presence of oedema (defining kwashiorkor) were used to pragmatically identify the malnourished children [21]. Two time periods were considered: April 2002 - April 2006 cohort which included 954 children treated with the previous quinine regimen (15 mg/kg intravenous loading dose and a maintenance dose of 10 mg/kg 12-hourly) diluted in 5% dextrose and infused intravenously over 2 hours; and May 2006 - July 2009 cohort which included 283 children treated with the new WHO recommended quinine regimen (20 mg/kg intravenous loading dose and a maintenance dose of 10 mg/kg 8-hourly) diluted in 5% dextrose and infused intravenously over 4 hours. Quinine used in both periods was obtained from a single supplier (Indus Pharma Ltd, Pakistan) together with their respective certificates of analysis for each batch.

Standard treatment included intravenous maintenance fluids (5% dextrose and 0.18% saline at 4 mls/kg/hour), intravenous quinine, and antibiotics. Appropriate treatment was started immediately after admission. This current study did not include any child admitted to the general ward with malaria parasitaemia who deteriorated after admission fulfilling the definition of severe malaria, albeit relatively few. Parenteral drugs and intravenous fluids were prescribed until the child was able to take and retain oral fluids and medication. Oral anti-malarial medication prescribed depended on the national treatment policy at the time and included sulfadoxine pyrimethamine (2002-2005) or artemether-lumefantrine (2006-2009). At admission blood glucose was measured in the laboratory using an automated glucose oxidase system (Analox instrument (Analox(r) London, UK)). Thereafter blood glucose was routinely monitored using the bedside monitoring device Accu-Chek™ Advantage (Roche diagnostics, Sydney, Australia; precision of 0.1 mmolL) at one hour post admission and four-hourly until cessation of quinine. Hypoglycaemia, defined as a blood glucose less than or equal to 3 mmol, determined by either device, was managed with an intravenous bolus of 5 ml/kg of 10% dextrose which has been the standard of care in the HDU since 2000 [18]. Additional measurements were conducted during episodes of seizure or posturing activity, transfusion or clinical deterioration. The Accu-Chek™ machine was calibrated weekly and every time a new box of test strips was opened.

In-patient medical and nursing case notes and observation charts for all children admitted with severe malaria over the period of the study were reviewed to identify and confirm episodes of hypoglycaemia (capillary or venous blood glucose equal or less than 3.0 mmol/l) that occurred post-admission and therefore after commencement of parenteral quinine. Clinical events occurring around each hypoglycaemia episode (4 hrs before and one hour after the episode) were examined and recorded. We also examined the frequency of hypoglycaemia cases for three different definition thresholds; < 2.2 mmol/l (severe hypoglycaemia), < 2.5 mmol/l (moderate hypoglycaemia), and < = 3 mmol/l (any hypoglycaemia). In clinical practice different blood glucose thresholds are used to define hypoglycaemia. Studies have shown that hypoglycaemia < 2.2 mmols/L is associated with poor outcome [22] and this complication is incorporated within the case definition of severe malaria in children and adults [23]. International paediatric guidelines recognize a blood glucose of < 3 mmols/L, in children with severe disease as a therapeutic indication for treatment [24]. We adopted this practice in 2002 for all severely ill children. The WHO guidelines, on the other hand, recommends < 2.5 mmol/l threshold for treatment of hypoglycaemia in children with severe illness [18].

There was incomplete data on the prevalence of neurological sequelae after discharge and therefore this data was not included.

### Statistical analysis

Baseline characteristics were compared between the two groups (regimens). Proportions were compared for categorical variables using Chi-square tests and Fisher's exact where appropriate. Malaria parasitaemia was summarised as a geometric mean [95% reference range]. For continuous variables, Student's t-test was used to compare mean differences between the two regimens. In any case of non-normal data, Mann Whitney (Wilcoxon Rank sum) test was used to make comparison between the two groups. Relative risk of hypoglycaemia in the two treatment groups was also estimated. Clinical definitions of severity features and our definition of circulatory collapse (hypovolaemic shock) have been described previously [13,23]. Variables with clinical plausibility or that were imbalanced at baseline between the two groups were chosen for univariable analysis to test association with post-admission hypoglycaemia. Those variables with p-values ≤ 0.15 were chosen for inclusion in the multivariable logistic regression to adjust for confounding. Variables were adjusted for age in the multiple regression analysis. The regression model was tested for specification error and goodness of fit. Significance was evaluated at 5% level. Analysis was done using Stata version 11 (Stata corporation, Texas, United States).

## Results

A total of 954 children were treated with the old regimen (April 2002 to April 2006) and 283 children were treated with the new regimen (May 2006 - July 2009). Male children comprised 50% of cases in both cohorts. Children treated with the old regimen cohort were younger, median age 28 months (IQR 18 - 43) compared to the new regimen 36 months (IQR 25 - 48), p < 0.01. Detailed admission characteristics for the children in two cohorts are shown in Table 1. Children in the 2006-2009 cohort had higher parasitaemia but a lower proportion of severity indicators (coma, deep breathing, severe anaemia and hypoglycaemia) than the 2002- 2006 cohort (Table 1). Severe hypoglycaemia (< 2.2 mmols/L) was also more common at admission in the 2002-2006 cohort than in the 2006-2009 cohort: 119 (12%) versus 19 (7%) (p = 0.007), respectively.

**Table 1:** Admission characteristics for children in the two cohorts

Clinical features	Quinine 15 mg/kg (N = 954) n (%)	Quinine 20 mg/kg (N = 283) n (%)	P-value ^Ŧ^
Coma (BCS ≤ 2)	434/953 (46)	123/279 (44)	0.67
Deep breathing	391/953 (41)	95/277 (34)	0.04
Impaired consciousness	706/953 (74)	204/279 (73)	0.91
Severe anaemia (Hb < 5 g/dl)	229/949 (24)	47/279 (17)	0.01
Temperature gradient	263/951 (28)	55/277 (20)	0.009
Delayed capillary refill (> 2 seconds)	232/953 (24)	41/279 (15)	0.001
Weak Pulse volume	159/953 (17)	31/277 (11)	0.03
Mid Upper Arm Circumference < 12 cm	59/914 (6)	9/240 (4)	0.11
Visible wasting	58/953 (6)	6/277 (2)	0.01
Seizures	168/953 (18)	38/277 (14)	0.13
Prostration	436/953 (46)	104/279 (37)	0.05
Hypoglycaemia < = 3 mmol/l	155/952 (16)	26/282 (9)	0.003
Severe hypoglycaemia(< 2.2 mmol/l)	118/952 (12)	19/282 (7)	0.008
Creatinine > 80 mmol/l	225/904 (25)	49/211 (23)	0.61
Potassium > 5 mmol/l	149/934 (16)	17/259 (7)	< 0.01
**Mean laboratory variable [SD]**			
Haemoglobin level, mg/dl	7.06 [2.63]	7.53 [2.44]	0.004
pH	7.28 [0.14]	7.30 [0.51]	0.34
pCO_2_,mmol/L	4.17 [1.92]	3.98 [1.73]	0.18
Base Deficit, mmol/L	9.99 [6.63]	8.49 [5.72]	0.002
Sodium level, mmol/l	134.33 [5.62]	133.39 [5.51]	0.02
Creatinine level, mmol/l	68.89[41.7]	66.81 [32.65]	0.49
Parasitaemia ×10^3^parasites/μl	3651 [3149 - 4234]	5604 [4393 - 7150]	0.01

IQR - Inter-quartile range, SE-Standard error, ^Ŧ ^Test statistic - χ^2 ^test or t-test on means

### Frequency of hypoglycaemia post admission

**Figure 1:**
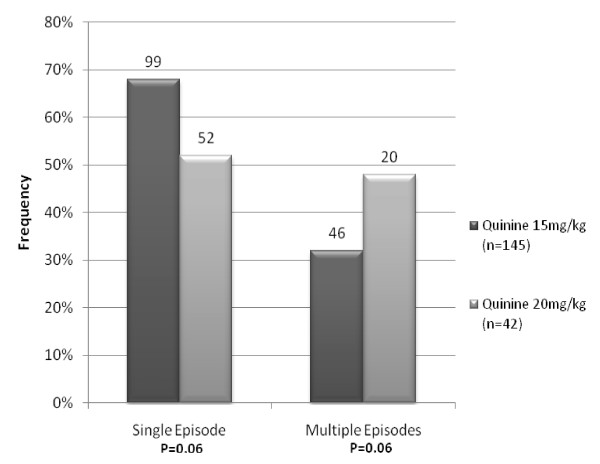
**Frequency of episodes in the two cohorts: Quinine 15 mg/kg n = 145, Quinine 20 mg/kg n = 42**.

Fifteen percent (145/954) of the 2002-2006 cohort developed hypoglycaemia (< = 3 mmols/l) post admission as did 15% (42/283) of the 2006-2009 cohort OR = 0.97, 95% confidence interval [0.67-1.41]; P = 0.88. The prevalence of hypoglycaemia, if compared with baseline levels, increased overall by 6% (from 9% at admission) in the 2006-2009 cohort, but decreased by 1% in the 2002-2006 cohort post admission (from 16% at admission). Of the 145 children in the 2002-2006 cohort who developed hypoglycaemia after treatment, 103 (71%) were euglycaemic at admission and 42 (29%) had hypoglycaemia at baseline. Sixty eight percent (99/145) of these children had a single episode and 46 (32%) had multiple episodes. Of the 42 children in the 2006-2009 cohort who developed hypoglycaemia after treatment, 27 (66%) were euglycaemic at admission whilst 14 (34%) had hypoglycaemia at admission. Fifty two percent (22/42) of the children had a single episode whilst the remaining 20 (48%) had multiple episodes. Overall, children presenting with hypoglycaemia at admission had increased odds of having an episode post admission, OR = 3.18 (CI 2.21 - 4.58, p < 0.0001). For children who were euglycaemic at admission there was no statistical difference in frequency of subsequent episodes between the two cohorts (p = 0.32). When comparing the numbers with multiple episodes of hypoglycaemia post admission between the two cohorts, there was weak evidence of a slight increase during the new regime (p = 0.06). Figure 1 shows the frequency of single and multiple episodes in the two cohorts.

### Timing and distribution of hypoglycaemia episodes

The distribution and timing of hypoglycaemia episodes was explored and the results are shown on Table 2. We found no statistical difference in the timing of the events between the two cohorts (χ^2 ^for trend, p = 0.15) although the children who developed hypoglycaemia receiving the new regimen (2006-2009 cohort) had a higher proportion of the first episode occurring after 24 hours (OR: 0.57; 95% CI: [0.26 - 1.24]).

**Table 2:** Time of occurrence of first hypoglycaemia episode and hypoglycaemia severity

	2002-2006 cohort: Quinine 15 mg/kg regimen		2006-2009 cohort: Quinine 20 mg/kg regimen	
Time period	Frequency by first episode (n = 145) n (%)	Frequency by all episodes (n = 229) n (%)	Frequency by first episode (n = 42) n (%)	Frequency by all episodes (n = 86) n (%)
0 - 24 hrs	118 (81)	157 (69)	30 (71)	47 (55)
After 24 hrs	19 (13)	41 (18)	6 (14)	21 (24)
After 48 hrs	8 (6)	31 (14)	6 (14)	18(21)
**Severity (Blood glucose levels), n/N (%)**				
< 2.2 mmol/l	74/954 (8)		13/283(5)	P = 0.07
< 2.5 mmol/l	103/954 (11)		22/283(8)	P = 0.14
< = 3.0 mmol/l	145/954 (15)		42/283(15)	P = 1.00
**Final outcome (death) - those with and those without any hypoglycaemia post admission, n/N (%)**				
Hypoglycaemia = Yes	29/145 (20)		15/42 (36)	P = 0.04
Hypoglycaemia = No	73/809 (9)		13/240 (5)	P = 0.07
**Final outcome (death) - those with and those without any hypoglycaemia at or during admission, n/N (%)**				
Hypoglycaemia = Yes	45/155 (29)		11/26 (42)	P = 0.18
Hypoglycaemia = No	57/797 (7)		17/255 (7)	P = 1.00

### Severity of Hypoglycaemia

**Figure 2:**
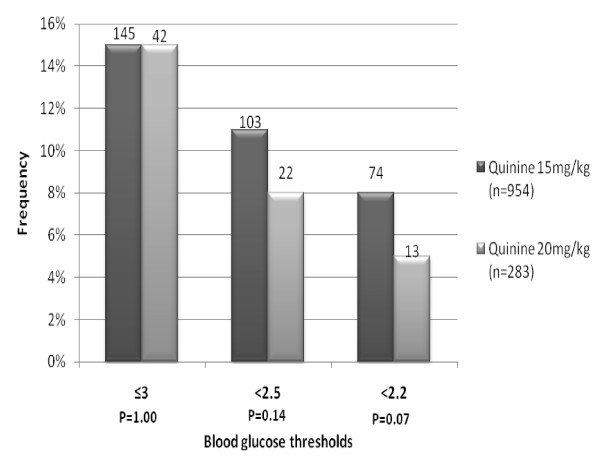
**Frequency of hypoglycaemia severity using different definitions**. The x-axis includes blood sugar thresholds of < = 3 mmols/L, 2.5 mmols/L and < 2.2 mmols/L

Using the strict definition for severe hypoglycaemia (< 2.2 mmol/l) a lower proportion of children developed profound hypoglycaemia, 13/283 (5%) on the new regimen group compared to the old regimen group (2002-2006 cohort), 74/954 (8%) (OR: 0.57; 95% CI: [0.32 - 1.04], P = 0.07). Using a slightly less stringent threshold (< 2.5 mmol/l) we found little difference in the numbers developing hypoglycaemia (22/283, 8%) on new regimen compared to (103/954, 11%) on the old regimen (OR: 0.70; 95% CI: [0.43 - 1.12], P = 0.14). Table 2 summarises the distribution and severity of the episodes of hypoglycaemia post admission and Figure 2 shows the frequency of hypoglycaemia using the different cut-offs in the two cohorts.

### Mortality and Clinical associations

In-hospital case fatality was similar in the respective cohorts, 102/954 (11%) and 28/283 (10%) (χ^2 ^= 0.13, p = 0.71). For both cohorts the development of any episode of hypoglycaemia (< = 3 mmols/L) after admission was associated with an increased odds of dying when compared to those who remained euglycaemic, 44/187 (24%) and 86/1049 (8%) respectively, OR = 3.45 (95% confidence interval = 2.30-5.16), (P < 0.01).

Clinical events concurring with episodes of hypoglycaemia in both cohorts included coma (defined as BCS < = 2) in 57% of the episodes; evidence of circulatory failure (hypovolaemic shock) in 38% of episodes; 21% were associated with clinical features of metabolic acidosis (deep breathing). In addition, 24% (45/187) of episodes occurred during a blood transfusion whilst 37% (70/187) of the episodes occurred during a period of inadequate glucose supply (accidental or actual discontinuity of intravenous maintenance glucose). Episodes of seizures and/or posturing were less frequently associated with hypoglycaemia; they were co-incident in 10% and 9% of the hypoglycaemia episodes, respectively. Our preliminary exploration of the data which examined only the first episode of hypoglycaemia (< = 3 mmols/L) after admission indicated little differences in the frequency of these associated clinical factors between the two cohorts.

### Predictors of hypoglycaemia post admission

In the age-adjusted analysis of the predictors of hypoglycaemia post admission, the following factors were associated with increased risk of developing hypoglycaemic post admission: hypoglycaemia at admission, deep breathing, severe anaemia, temperature gradient (a sign of impaired perfusion and pragmatically assessed by running the back of the hand from the foot up the shin: where the foot was cooler than the shin this indicates a temperature gradient) and weak pulse volume (an advanced sign of hypovolaemic shock) (Table 3). Multivariable logistic regression was used to adjust for variables that were predictive in the univariable analysis (variables with p values < 0.15). The following variables were significant predictors of hypoglycaemia post admission at multiple regression analysis: hypoglycaemia at admission, severe anaemia and temperature gradient (all adjusted for age in months).

**Table 3:** Predictors of Hypoglycaemia post admission

	Univariable analysis		Multiple regression analysis	
	Unadjusted OR [CI]	P-Value	Adjusted OR [CI]*	P-Value
**Treatment:Regimen**	0.97 [0.67 - 1.41]	0.88	-	-
**Hypoglycaemia at admission**	3.18 [2.21 - 4.58]	< 0.001	2.76 [1.87 - 4.06]	< 0.001
**Deep breathing**	1.46 [1.06 - 1.99]	0.02	0.96 [0.67 - 1.36]	0.80
**Severe anaemia**	1.91 [1.36 - 2.69]	< 0.001	1.57 [1.08 - 2.29]	0.02
**Temperature Gradient**	2.10 [1.51 - 2.92]	< 0.001	1.77 [1.22 - 2.57]	0.003
**Delayed Capillary refill time**	1.63 [1.15 - 2.31]	0.006	1.00 [0.65 - 1.55]	0.98
**Visible wasting**	1.78 [0.98 - 3.25]	0.06	1.32 [0.69 - 2.54]	0.40
**Prostration**	0.88 [0.64 - 1.20]	0.41	-	-
**Weak pulse volume**	1.63 [1.11 - 2.41]	0.01	0.93 [0.58 - 1.49]	0.75
**Parasitaemia^#^**	1.29 [0.86 - 1.95]	0.22	-	-

*Adjusted for age in months; OR - Odds ratio; CI - Confidence Interval.

Treatment, prostration and parasitaemia not included in the multivariable model as p > 0.15

^#^Parasitaemia was categorised as below or above 5000 parasites/microlitre

## Discussion

Despite some recent and localized successes in disease control severe malaria remains a major public health burden in sub Saharan Africa killing over half million children each year [23]. Parenteral quinine is therefore one of the most widely used treatment in African hospitals and information about the potential iatrogenic side effects remain pertinent. Our study provides valuable information for clinicians treating children with severe malaria in Africa with respect to the occurrence of life-threatening hypoglycaemia and its clinical associations. Hypoglycaemia occurs in 15% of children following admission, with over 70% of episodes occurring within 24 hours of admission. We found no evidence of a dose-response relationship between quinine and any degree of hypoglycaemia after admission. The most important clinical variables at admission that predicted development of hypoglycaemia after admission were hypoglycaemia, severe anaemia and temperature gradient (a marker of impaired perfusion or circulatory failure). Most episodes were single events and frequently concurrent with one or more markers of disease severity including coma, clinical features of impaired perfusion, deep breathing (Kussmaul breathing) but less commonly associated with the time-honoured suspected clinical associations of seizures or posturing. We found a coincidence of hypoglycaemic episodes during transfusions or periods of disruption of intravenous glucose infusion. Overall, fatal outcome was more common in children developing hypoglycaemia after admission than their euglycaemic counterparts.

Our previous regime, based upon pharmacokinetic studies, indicated that lower doses were warranted due to a smaller apparent volume of distribution in the children, and that a higher dosage regime was justifiable for adults and in areas where quinine-resistance was more problematic [19]. A recent large trial in Asia (SEQUAMAT) showed that parenteral artesunate reduced mortality by 35% compared to conventional quinine treatment [25]. SEQUAMAT, together with previous studies, have shown a lower frequency of hypoglycaemia in the artesunate treated group [26-29]. Our data, and that of previous studies, do not suggest any dose- dependent relationship with quinine treatment in African children with severe malaria. For this and many other reasons a separate trial in African children is underway (AQUAMAT ISRCTN 50258054).

Hypoglycaemia remains a common complication of severe malaria, occurring throughout the clinical course. The origin, in African children, is unlikely to be iatrogenic. Studies comparing children with severe malaria and other severe illnesses found no difference in the frequency of this complication, indicating that it is a consequence of multi-organ involvement manifest in severe life-threatening illness [11]. Plasma glucose concentration reflects the balance between glucose supply and glucose use in children with severe illness including malaria. Inadequate glucose supply and fasting have been implicated in a number of studies in children with severe malaria [11,30] but others examining glucose kinetics conclude that gluconeogenesis and endogenous glucose production is preserved or even increased [28,31] but glucose supply however, is disproportionately derived from glycogenolysis. Zijlmans and colleagues recently demonstrated that even after 16 hours of fasting glycogenolysis contributed to 35-40% of the glucose production [32]. This finding is surprising given that it had been previously assumed that the glycogen stores in young children are believed to support a maximum fasting period of 12 hours [33]. In our study we found that discontinuation of a 5% glucose infusion either prematurely in a fasting child, during a transfusion or accidentally (non-patency of an intravenous cannula) was one of the events that were commonly observed in association with hypoglycaemia. Nutritional status is an important determinant of glycogen stores, which may be critical in a severely ill child. Most of our cohort had mild to moderate undernutrition, with the median weight for height or length z-score (WHZ-score) -1.5 [IQR (-2.4, -0.65)], which may explain why fasting was associated with early hypoglycaemia (within the four hours period of the event) and at variance with the studies in Surinamese children, whose median WHZ-score was 0.6 [32]. These observations concur with our earlier studies indicating that whilst glucose production is increased in severe malaria it fails to compensate to the increased demand and therefore puts children at risk of developing hypoglycaemia [30]. For children receiving blood transfusion, which ought to contain adequate dextrose/citrate to prevent hypoglycaemia, either the stress of transfusion or relative inadequacy of glucose supply may lead to a higher risk of hypoglycaemia in a severely ill child. Whilst inappropriate transfusions should be discouraged we strongly recommend additional glucose monitoring of children receiving blood transfusion.

The characteristic clinical manifestations of hypoglycaemia are rarely evident owing to disease severity and careful monitoring is recommended to facilitate early diagnosis and prevent poor outcome and neurological sequelae [34-36]. There is need for more evidence from randomised trials to define the ideal threshold for treatment of hypoglycaemia as recent observational studies have shown that mortality is still higher at a threshold greater than 3.0 mmol/l. A recent review by Achoki and colleagues demonstrated in a prospective cohort study of all paediatric admissions that blood glucose concentrations at admission of up to 4.0 mmol/l was associated with increased odds of mortality compared to all levels of glucose concentration above this threshold [37]. Another recent study conducted in Mali by Willcox and colleagues demonstrated that low glycaemia (2.2-4.4 mmol/l) was significantly associated with odds of mortality in children with a clinical diagnosis of severe malaria [38]. The authors concluded that the optimum threshold is 6.1 mmol/l for predicting mortality which is much higher than current definitions.

Our study has a number of limitations. First, the retrospective design, rather than a randomised trial, meant that the two study cohorts were not identical in terms of overall disease-severity indices. The children in the 2006- 2009 cohort were on average older, had fewer severity indicators at admission but survival was similar in the two groups. The clinical associations with hypoglycaemia after admission were identical and reassuringly the occurrence of severe hypoglycaemia was less apparent in the latter cohort. Secondly, glycaemic control was monitored using bedside handheld glucometers. The performance of point of care devices in accurately determining hypoglycaemia has been questioned [39], since they have been associated with underestimation of hypoglycaemic events, particularly in patients with moderate to severe anaemia. Recent studies have demonstrated that moderate to severe anaemia explains most of the major variance between glucometer and quality control laboratory readings [40]. For patients with a haematocrit below 20 the percentage error of glucometer measurements, compared to the reference laboratory glucose, was nearly 30% [40]. In our study, monitoring was largely by hand held devices, although this may have introduced a systematic error in glucose readings, the glucometers used in both cohorts were identical and therefore unlikely to have biased the comparison. Another limitation was the unequal numbers in the two cohorts. There were fewer cases of severe malaria in the 2006 - 2009 cohorts and this reflects the changing trend in malaria epidemiology in the study area [41].

## Conclusions

We found no relationship between quinine dosage and the frequency or severity of hypoglycaemia in African children with severe malaria. Overall 15% of children with severe malaria developed hypoglycaemia after admission most commonly on the day of admission. Hypoglycaemia occurrence was temporally associated with markers of disease severity and disruption in the maintenance glucose supply. Owing to the poor outcome a case fatality of 24% compared to 8% in euglycaemia children indicate the need for targeted glucose monitoring in children who remain severely ill and prevent premature termination of glucose maintenance infusion. Further research, including controlled trials, to establish best practice is urgently needed.

## Competing interests

The authors declare that they have no competing interests.

## Authors' contributions

GO, SA and KM - conceived and designed the study, acquisition of data, data analysis, drafting the manuscript and interpretation of data. JJ, GF - study design, data analysis, drafting the manuscript and interpretation of the data. MB and EK- conceived and designed the study, acquisition of data and interpretation of data. All the authors read and approved the final manuscript.

## Pre-publication history

The pre-publication history for this paper can be accessed here:

http://www.biomedcentral.com/1471-2334/10/334/prepub

## References

[B1] White NJ Warrell DA Chanthavanich P Looareesuwan S Warrell MJ Krishna S Williamson DH Turner RC Severe hypoglycemia and hyperinsulinemia in falciparum malaria N Engl J Med 1983 309 2 61 66 10.1056/NEJM198307143090201 6343877

[B2] Elusiyan JB Adejuyigbe EA Adeodu OO Hypoglycaemia in a Nigerian paediatric emergency ward Journal of tropical pediatrics 2006 52 2 96 102 10.1093/tropej/fmi068 16169861

[B3] Taylor TE Molyneux ME Wirima JJ Fletcher KA Morris K Blood glucose levels in Malawian children before and during the administration of intravenous quinine for severe falciparum malaria N Engl J Med 1988 319 16 1040 1047 10.1056/NEJM198810203191602 3050516

[B4] Krishna S Waller DW ter Kuile F Kwiatkowski D Crawley J Craddock CF Nosten F Chapman D Brewster D Holloway PA Lactic acidosis and hypoglycaemia in children with severe malaria: pathophysiological and prognostic significance Trans R Soc Trop Med Hyg 1994 88 1 67 73 10.1016/0035-9203(94)90504-5 8154008

[B5] English M Wale S Binns G Mwangi I Sauerwein H Marsh K Hypoglycaemia on and after admission in Kenyan children with severe malaria Q J Med 1998 91 3 191 197 10.1093/qjmed/91.3.191 9604071

[B6] Marsh K Forster D Waruiru C Mwangi I Winstanley M Marsh V Newton C Winstanley P Warn P Peshu N Indicators of life-threatening malaria in African children N Engl J Med 1995 332 21 1399 1404 10.1056/NEJM199505253322102 7723795

[B7] Holding PA Stevenson J Peshu N Marsh K Cognitive sequelae of severe malaria with impaired consciousness Trans R Soc Trop Med Hyg 1999 93 5 529 534 10.1016/S0035-9203(99)90368-1 10696414

[B8] Idro R Jenkins NE Newton CR Pathogenesis, clinical features, and neurological outcome of cerebral malaria Lancet neurology 2005 4 12 827 840 10.1016/S1474-4422(05)70247-7 16297841

[B9] Schellenberg D Menendez C Kahigwa E Font F Galindo C Acosta C Schellenberg JA Aponte JJ Kimario J Urassa H African children with malaria in an area of intense Plasmodium falciparum transmission: features on admission to the hospital and risk factors for death Am J Trop Med Hyg 1999 61 3 431 438 10497986 10.4269/ajtmh.1999.61.431

[B10] Waller D Krishna S Crawley J Miller K Nosten F Chapman D ter Kuile FO Craddock C Berry C Holloway PA Clinical features and outcome of severe malaria in Gambian children Clin Infect Dis 1995 21 3 577 587 8527547 10.1093/clinids/21.3.577

[B11] Kawo NG Msengi AE Swai AB Chuwa LM Alberti KG McLarty DG Specificity of hypoglycaemia for cerebral malaria in children Lancet 1990 336 8713 454 457 10.1016/0140-6736(90)92009-7 1974988

[B12] Krishna S Waller DW ter Kuile F Kwiatkowski D Crawley J Craddock CF Nosten F Chapman D Brewster D Holloway PA Lactic acidosis and hypoglycaemia in children with severe malaria: pathophysiological and prognostic significance Trans R Soc Trop Med Hyg 1994 88 1 67 73 10.1016/0035-9203(94)90504-5 8154008

[B13] Maitland K Levin M English M Mithwani S Peshu N Marsh K Newton CR Severe P. falciparum malaria in Kenyan children: evidence for hypovolaemia Qjm 2003 96 6 427 434 10.1093/qjmed/hcg077 12788961

[B14] Roe JK Pasvol G New developments in the management of malaria in adults Qjm 2009 102 10 685 693 10.1093/qjmed/hcp087 19656846

[B15] Phillips RE Looareesuwan S Molyneux ME Hatz C Warrell DA Hypoglycaemia and counterregulatory hormone responses in severe falciparum malaria: treatment with Sandostatin Q J Med 1993 86 4 233 240 8327638

[B16] Das BS Satpathy SK Mohanty D Mohanty S Mishra SK Satapathy PC Patnaik JK Bose TK Hypoglycaemia in severe falciparum malaria Trans R Soc Trop Med Hyg 1988 82 2 197 201 10.1016/0035-9203(88)90407-5 3055451

[B17] White NJ Miller KD Marsh K Berry CD Turner RC Williamson DH Brown J Hypoglycaemia in African children with severe malaria Lancet 1987 1 8535 708 711 10.1016/S0140-6736(87)90354-0 2882130

[B18] WHO Pocket book of hospital care for children:guidelines for management of common illnesses with limited resources 2005 24006557

[B19] Winstanley P Newton C Watkins W Mberu E Ward S Warn P Mwangi I Waruiru C Pasvol G Warrell D Towards optimal regimens of parenteral quinine for young African children with cerebral malaria: the importance of unbound quinine concentration Trans R Soc Trop Med Hyg 1993 87 2 201 206 10.1016/0035-9203(93)90494-B 8337730

[B20] Winstanley PA Mberu EK Watkins WM Murphy SA Lowe B Marsh K Towards optimal regimens of parenteral quinine for young African children with cerebral malaria: unbound quinine concentrations following a simple loading dose regimen Trans R Soc Trop Med Hyg 1994 88 5 577 580 10.1016/0035-9203(94)90170-8 7992345

[B21] Berkley J Mwangi I Griffiths K Ahmed I Mithwani S English M Newton C Maitland K Assessment of severe malnutrition among hospitalized children in rural Kenya: comparison of weight for height and mid upper arm circumference Jama 2005 294 5 591 597 10.1001/jama.294.5.591 16077053

[B22] White NJ Miller KD Marsh K Berry CD Turner RC Williamson DH Brown J Hypoglycaemia in African children with severe malaria Lancet 1987 1 8535 708 711 10.1016/S0140-6736(87)90354-0 2882130

[B23] Severe falciparum malaria. World Health Organization, Communicable Diseases Cluster Trans R Soc Trop Med Hyg 2000 94 Suppl 1 S1 90 11103309

[B24] Group ALS Advanced Paediatric Life Support: The Practical Approach 2004 4 WileyBlackwell

[B25] Dondorp A Nosten F Stepniewska K Day N White N Artesunate versus quinine for treatment of severe falciparum malaria: a randomised trial Lancet 2005 366 9487 717 725 10.1016/S0140-6736(05)67176-0 16125588

[B26] Newton PN Angus BJ Chierakul W Dondorp A Ruangveerayuth R Silamut K Teerapong P Suputtamongkol Y Looareesuwan S White NJ Randomized comparison of artesunate and quinine in the treatment of severe falciparum malaria Clin Infect Dis 2003 37 1 7 16 10.1086/375059 12830403

[B27] Davis TM Binh TQ Thu le TA Long TT Johnston W Robertson K Barrett PH Glucose and lactate turnover in adults with falciparum malaria: effect of complications and antimalarial therapy Trans R Soc Trop Med Hyg 2002 96 4 411 417 10.1016/S0035-9203(02)90377-9 12497978

[B28] Agbenyega T Angus BJ Bedu-Addo G Baffoe-Bonnie B Guyton T Stacpoole PW Krishna S Glucose and lactate kinetics in children with severe malaria J Clin Endocrinol Metab 2000 85 4 1569 1576 10.1210/jc.85.4.1569 10770199

[B29] Woodrow CJ Planche T Krishna S Artesunate versus quinine for severe falciparum malaria Lancet 2006 367 9505 110 111 author reply 111-112 10.1016/S0140-6736(06)67957-9 16413871

[B30] Dekker E Hellerstein MK Romijn JA Neese RA Peshu N Endert E Marsh K Sauerwein HP Glucose homeostasis in children with falciparum malaria: precursor supply limits gluconeogenesis and glucose production J Clin Endocrinol Metab 1997 82 8 2514 2521 10.1210/jc.82.8.2514 9253327

[B31] Atabani GS Saeed BO elSeed BA Bayoumi MA Hadi NH Abu-Zeid YA Bayoumi RA Hypoglycaemia in Sudanese children with cerebral malaria Postgraduate medical journal 1990 66 774 326 327 10.1136/pgmj.66.774.326-a 2201015 PMC2429396

[B32] Zijlmans W van Kempen A Ackermans M de Metz J Kager P Sauerwein H Glucose kinetics during fasting in young children with severe and non-severe malaria in Suriname Am J Trop Med Hyg 2008 79 4 605 612 18840752

[B33] Osier FH Berkley JA Ross A Sanderson F Mohammed S Newton CR Abnormal blood glucose concentrations on admission to a rural Kenyan district hospital: prevalence and outcome 2003 88 12818911 10.1136/adc.88.7.621 PMC1763181

[B34] Bondi FS The incidence and outcome of neurological abnormalities in childhood cerebral malaria: a long-term follow-up of 62 survivors Trans R Soc Trop Med Hyg 1992 86 1 17 19 10.1016/0035-9203(92)90420-H 1566292

[B35] Holding PA Stevenson J Peshu N Marsh K Cognitive sequelae of severe malaria with impaired consciousness Trans R Soc Trop Med Hyg 1999 93 5 529 534 10.1016/S0035-9203(99)90368-1 10696414

[B36] Idro R Carter JA Fegan G Neville BG Newton CR Risk factors for persisting neurological and cognitive impairments following cerebral malaria Arch Dis Child 2006 91 2 142 148 10.1136/adc.2005.077784 16326798 PMC2082712

[B37] Achoki R Opiyo N English M Mini-review: Management of Hypoglycaemia in Children Aged 0-59 Months J Trop Pediatr 2009 19933785 10.1093/tropej/fmp109 PMC2948531

[B38] Willcox ML Forster M Dicko MI Graz B Mayon-White R Barennes H Blood glucose and prognosis in children with presumed severe malaria: is there a threshold for 'hypoglycaemia'? Trop Med Int Health 15 2 232 240 10.1111/j.1365-3156.2009.02444.x 19961563

[B39] Mann EA Pidcoke HF Salinas J Wade CE Holcomb JB Wolf SE Accuracy of glucometers should not be assumed Am J Crit Care 2007 16 6 531 532 author reply 532 17962493

[B40] Pidcoke HF Wade CE Mann EA Salinas J Cohee BM Holcomb JB Wolf SE Anemia causes hypoglycemia in intensive care unit patients due to error in single-channel glucometers: methods of reducing patient risk Crit Care Med 2010 38 2 471 476 10.1097/CCM.0b013e3181bc826f 19789438 PMC4267684

[B41] O'Meara WP Bejon P Mwangi TW Okiro EA Peshu N Snow RW Newton CR Marsh K Effect of a fall in malaria transmission on morbidity and mortality in Kilifi, Kenya Lancet 2008 372 9649 1555 1562 10.1016/S0140-6736(08)61655-4 18984188 PMC2607008

